# An exclusion mechanism is epistatic to an internal detoxification mechanism in aluminum resistance in Arabidopsis

**DOI:** 10.1186/s12870-020-02338-y

**Published:** 2020-03-18

**Authors:** Yuqi Wang, Wancong Yu, Yu Cao, Yanfei Cai, Sangbom M. Lyi, Weiwei Wu, Yan Kang, Cuiyue Liang, Jiping Liu

**Affiliations:** 1 Key Laboratory for Water Quality and Conservation of the Pearl River Delta, Ministry of Education, School of Environmental Science and Engineering, Guangzhou University, Guangzhou, 510006 Guangdong China; 2 Robert Holley Center, US Department of Agriculture, Agricultural Research Service, Cornell University, Ithaca, NY 14853 USA; 3 Medical Plant Laboratory, Tianjin Research Center of Agricultural Biotechnology, Tianjin, 300384 China; 4 College of Natural Resources and Environment, South China Agricultural University, Guangzhou, 510642 Guangdong China; 5 School of Life Sciences, Inner Mongolia University, Hohhot, 010021 Inner Mongolia China

**Keywords:** ALMT1, Epistasis, Malate, NIP1;2, Organic acid, Resistance mechanism, Root cell wall

## Abstract

**Background:**

In *Arabidopsis*, the aluminum (Al) exclusion mechanism is mainly facilitated by ALMT1-mediated malate exudation and MATE-mediated citrate releases from the root. Recently, we have demonstrated that coordinated functioning between an ALMT1-mediated Al exclusion mechanism, via exudation of malate from the root tip, and a NIP1;2-facilitated internal detoxification mechanism, via removal of Al from the root cell wall and subsequent root-to-shoot Al translocation, plays critical roles in achieving overall Al resistance. However, the genetic relationship between *ALMT1* and *NIP1;2* in these processes remained unclear.

**Results:**

Through genetic and physiological analyses, we demonstrate that unlike *ALMT1* and *MATE*, which function independently and additively, *ALMT1* and *NIP1;2* show an epistatic relationship in Al resistance. These results indicate that *ALMT1* and *NIP1;2* function in the same biochemical pathway, whereas *ALMT1* and *MATE* in different ones.

**Conclusion:**

The establishment of the epistatic relationship and the coordinated functioning between the ALMT1 and NIP1;2-mediated exclusion and internal detoxification mechanisms are pivotal for achieving overall Al resistance in the non-accumulating Arabidopsis plant. We discuss and emphasize the indispensable roles of the root cell wall for the implementation of the Al exclusion mechanism and for the establishment of an epistatic relationship between the ALMT1-mediated exclusion mechanism and the NIP1;2-facilitated internal detoxification mechanism.

## Background

Aluminum (Al) is the most abundant metal element in the earth crust [1]. Under neutral or alkalescent conditions, Al is present in the soil as forms that are non-toxic to plants [2]. However, at low pH (< 5.0), aluminum ions (Al^3+^) are dissolved and released from the soil clays into the soil solutions, which could interact with multiple sites of the plant root cell, including the cell wall, cell membrane and cytosol with toxic effects, resulting severe root growth inhibition, the most significant symptom of Al toxicity [1, 3]. The impaired root system restricts root absorption of water and nutrients from the acid subsoil, leading to drought stresses and nutrient deficiencies and thus reduced yields for crops grown on acidic soils [1, 4].

Physiologic studies have indicated that the root apex, rather than the root elongation zone and the mature root region, is a major target of Al toxicity [5]. Cell wall loosening and synthesis of cell wall components are essential for sustained root cell elongation and water uptake [6, 7]. However, Al^3+^ ions severely inhibit root elongation through reducing cell wall cation binding, water permeability and cell wall enzyme activities [8–11]. As a result, the root cell wall in the root apical region is one of the major targets for Al toxicity [12, 13].

Plants have adopted several strategies to cope with Al stresses, including Al exclusion and internal detoxification mechanisms [14, 15]. The exclusion mechanism relies on root releases of chemical exudates, including organic acids [16], phenolic compounds [17], and phosphate [18], which facilitates the formation of non-toxic Al-exudate complexes in the rhizosphere and thereby prevents Al from entering the root cell, including the root apoplast [19]. Aluminum exclusion via release of organic-acid anions, including malate, citrate and oxalate, from the root apex is the best characterized, the most effective and widespread resistance mechanism employed by a large number of monocots and dicots plants [16, 20–28]. Recently, Al- and salicylic acid (SA)-activated root exudation of benzoxazinoids has been recognized as an important exclusion mechanism for Al resistance in Maize [29].

The internal tolerance mechanism facilitates the detoxification of Al^3+^ internally [30]. The processes include chelation of Al by organic acids in the cytosol, Al compartmentation in the vacuole of the root cell, translocation from sensitive root tissues to less sensitive shoot tissues where it is further sequestrated into the vacuoles of shoot cells [30–32]. However, functional and regulatory components underlying these processes remain largely unclear.

In *Arabidopsis thaliana*, the exclusion mechanism plays a key role in Al resistance [24, 25], which mainly relies on Al-activated root exudation of malate and citrate via the plasma membrane (PM)-localized malate transporter, ALMT1, and the citrate transporter MATE from the multidrug and toxic compound extrusion family, respectively [24, 25]. ALMT1 facilitates the exudation of a large amount of malate from the root tip, while MATE mediates the release of a smaller amount of citrate from the more mature root region upon Al exposure [26]. The expression of both *ALMT1* and *MATE* is under the control of a master transcription factor, STOP1, i.e., sensitive to proton rhizotoxicity 1, which plays key roles in regulation of resistance to proton (low pH) and Al toxicity in plants [25, 33, 34].

In *Arabidopsis*, Al^3+^ ions in the rhizosphere can freely move and be retained in the root cell wall at low pH (< 5.0) [19]. The Al^3+^ ions in the root cell wall directly or indirectly activate the PM-localized malate and citrate transporters, leading to organic acid releases from the cytosol of the root cell and formation of Al-organic acid complexes in the rhizosphere as well as in the root cell wall. Although it has been demonstrated that Al-organic acid complexes in the rhizosphere cannot enter the root cell, including the root cell wall [19], whether the Al-organic acid complexes retained in the root cell wall are toxic to the plant remained unclear previously.

Our recent studies have demonstrated that the Arabidopsis nodulin 26-like intrinsic protein 1;2 (*NIP1;2*) gene encodes a PM-localized transporter that specifically transports Al-malate (Al-Mal) complexes but not charged Al^3+^ ions or other forms of Al-ligand complexes from the root cell wall into the root symplast [19, 35]. As the transport substrate of NIP1;2 is the Al-Mal complex but not the Al^3+^ ion, the ALMT1-mediated malate release into the root cell wall is a prerequisite for the NIP1;2-facilitated removal of Al from the root cell wall and subsequent translocation from the sensitive root tissues to the less sensitive shoot tissues [19]. Thus, the coordinated activities between the exclusion mechanism facilitated by ALMT1-mediated malate releases and the NIP1;2-mediated internal detoxification mechanism are essential for achieving overall Al tolerance in Arabidopsis [19, 35].

In genetics, the terms dominant and recessive are used to describe the effects of different alleles at a genetic locus on determining the expression of a trait. Dominant alleles (AA) ultimately determine the expression of the trait, whereas recessive alleles (aa) are much less likely to be expressed. When a dominant allele is paired with a recessive one (Aa), the dominant allele (A) determines the trait. Recessive traits only manifest when both alleles in the locus are recessive in an individual (aa). In contrast, the term of epistasis is used to describe interactions between genes located in different genetic loci (e.g., A and B). It is referred to as a situation where the allelic actions of one locus (i.e., AA, Aa, or aa) mask the allelic effects of another locus (i.e., BB, Bb and bb), in the same way where the dominant allele mask the effects of the recessive one at the same locus [36, 37]. In other words, epistasis describe a situation where the phenotypic expression at one locus depends on the genotype of a different locus.

Here, we provide further genetic evidence for the existence of an epistatic relationship between *ALMT1* and *NIP1;2*. We demonstrate that such an epistatic relationship is required for orchestrating the functions of different Al resistance mechanisms in Arabidopsis. We emphasize the essential role of the root cell wall in establishing the epistatic relationship between the ALMT1-mediated exclusion mechanism and the NIP1;2-facilitated internal detoxification mechanism in Arabidopsis. We also discuss possible relationships between the exclusion and the internal detoxification mechanisms for Al accumulating plants under Al stresses.

## Results

### Generation of an *amlt1_nip1;2* double mutant line

Three independent T-DNA insertion mutants of *NIP1;2*, i.e., *nip1;2–1* (SALK_126593), *nip1;2–2* (SALK_147353) and *nip1;2–3* (SALK_076128), displayed comparable hypersensitive phenotypes to Al stresses at pH 4.3 (Additional file 1: Figure S1) [19]. To further study the functional and genetic relationships between *NIP1;2* and *ALMT1*, a homozygous *almt1_nip1;2* double mutant line was generated through a cross between *almt1* (SALK_009629) and *nip1;2–3* (hereafter *nip1;2*), followed by selection from the F2 population of mutant plants with homozygous *almt1* and *nip1;2* alleles.

**Fig. 1:**
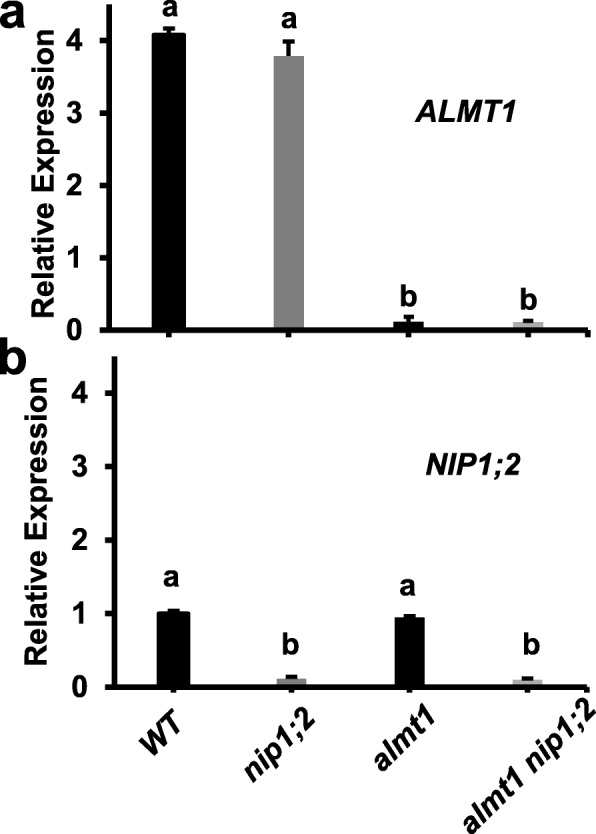
Expression patterns of *ALMT1* (**a**) and *NIP1;2* (**b**) in the root. Roots of 7-day-old seedlings of *WT*, *almt1, nip1;2* and *almt1_nip1;2* treated with 20 μM AlCl_3_ (pH 4.3) for 24 h were subject to RT-qPCR analysis. Data are means ± s.d. (*n* = 3); Different letters indicate significant differences between individual lines

Real-time reverse transcription-quantitative polymerase chain reaction (RT-qPCR) analyses indicated that in the wild type (WT, *Col-0*), the expression of *ALMT1* and *NIP1;2* in the root was both induced by Al treatment although the levels of *ALMT1* transcripts were about 4-fold higher than those of *NIP1;2* (Fig. 1). Under the *nip1;2* mutant background, the level of *ALMT1* expression in the root was comparable with that in the WT (Fig. 1a), whereas *NIP1;2* expression was greatly suppressed (Fig. 1b). In contrast, under the *almt1* background, although the level of the *NIP1;2* expression in the root were comparable with that in the WT, the Al-induced *ALMT1* expression in the root was barely detectable (Fig. 1a). These results confirmed that both *almt1* and *nip1;2* are knockout (KO) mutants [19, 24] and the expression of *ALMT1* and *NIP1;2* is independent of each other [35]. Under the *almt1_nip1;2* background, the expression of *ALMT1* and *NIP1;2* in the root was both barely detectable (Fig. 1a, b), indicating that *almt1_nip1;2* is a double KO mutant line.

### Comparable sensitivity between *almt1_nip1;2* and *almt1* to Al stresses

**Fig. 2:**
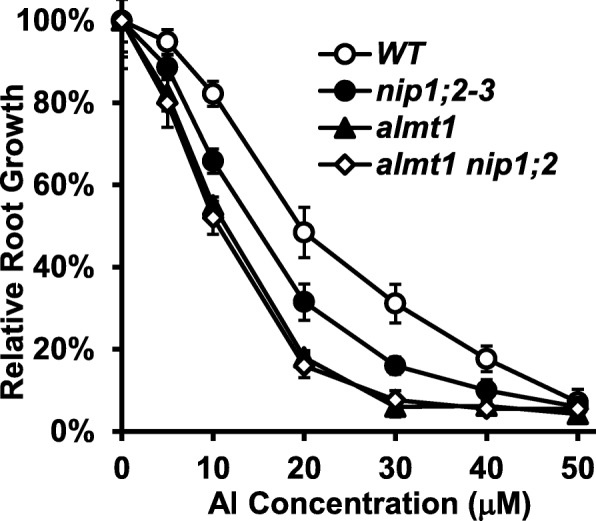
Relative root growth of *WT* and *almt1, nip1;2* and *almt1_nip1;2* mutants. Seeds were germinated and grown in hydroponic solution (pH 4.3) supplemented with 0, 5, 10, 20, 30, 40, 50 μM of AlCl_3_ for 5 days. Root length (mm) of individual seedlings was measured. Relative root growth (RRG%) was calculated according to the following formula: RRG% = root growth of individual plants under Al treatment/mean root growth under the control (−Al) condition. Data are means ± s.d. (*n* = 10)

To evaluate Al resistance of individual lines, relative root growth (RRG%) was calculated for 5-day-old plants of *WT*, *nip1;2*, *almt1* and *almt1_nip1;2* treated with a range of Al concentrations (0–50 μM) at pH 4.3 (Fig. 2). Root growth of both the *almt1* and *nip1;2* single mutants was more severely inhibited by Al than was the WT over the range of Al concentrations tested (Fig. 2). Moreover, root growth was more strongly inhibited in *almt1* than in *nip1;2* (Fig. 2). For instance, at Al concentration of 20 μM, root growth was inhibited by 82 and 69% in *almt1* and *nip1;2*, respectively (Fig. 2 and Additional file 1: Figure S2).

In contrast, no significant differences in root growth were observed between the *almt1_nip1;2* double mutant and the *almt1* single mutant over the entire range of Al concentrations tested (Fig. 2). Therefore, the Al-resistant phenotype of *almt1_nip1;2* resembled that of *almt1*, but not *nip1;2*.

### Genetic analysis of allelic effects of *ALMT1* and *NIP1;2* on Al resistance

To evaluate the effects of genotypic variations at the *ALMT1* and *NIP1;2* loci on Al resistance, a homozygous *almt1* plant was crossed with a homozygous *nip1;2* plant to generate heterozygous F1 plants. The dominant and recessive alleles of *ALMT1* and *NIP1;2* would be segregated among the F2 plants.

Root growth was measured for F2 seedlings germinated and grown for 7 days in a hydroponic growth solution (pH 4.3) supplemented with 20 μM AlCl_3_. Based on their root growth, the F2 plants could be classified into three phenotypic groups (A, B and C) that showed significant differences in root growth (mm) under Al treatment (Table 1 and Additional file 1: Figure S3). On the other hand, based on their allelic variations at the *ALMT1* and *NIP1;2* loci, the F2 population could be classified into nine distinct genotypic groups/combinations (Table 1 and Additional file 1: Figure S3).

**Table 1:** Allelic effects of *ALMT1* and *NIP1;2* on Al resistance

Genotypes at the ***ALMT1*** locus	Genotypes at the ***NIP1;2*** locus		
	*nip1;2/nip1;2*	*nip1;2/NIP1;2*	*NIP1;2/NIP1;2*
*almt1/almt1*	2.9 ± 0.5^**B**^	2.7 ± 0.9^**B**^	3.1 ± 0.7^**B**^
*almt1/ALMT1*	6.3 ± 1.4^**C**^	12.1 ± 2.4^**A**^	10.9 ± 1.6^**A**^
*ALMT1/ALMT1*	6.8 ± 1.8^**C**^	11.7 ± 1.9^**A**^	11.5 ± 2.8^**A**^

Seeds were germinated and grown in hydroponic growth medium supplemented with 20 μM AlCl_3_ (pH 4.3) for 7 days. Data are mean root length (mm) ± s.d. (*n* = 10). *Letters* represent groups with significant differences in root length (*P* ≤ 0.05) as determined by Fisher’s least significant difference (LSD) test.

Analysis of the relationship between phenotypic and genotypic variations indicated that *1)* the F2 plants in the phenotypic group A had at least one dominant wild-type allele at both the *ALMT1* and *NIP1;2* loci (Table 1 and Additional file 1: Figure S3); *2)* group B had homozygous *almt1/almt1* alleles regardless of the status of the *NIP1;2* alleles; and *3)* group C had homozygous *nip1;2/nip1;2* mutant alleles and at least one wild-type allele of *ALMT1*, i.e., *almt1/ALMT1* or *ALMT1/ALMT1*.

In phenotypic group A, plants with at least one wild-type *ALMT1* allele and one wild-type *NIP1;2* allele had comparable root growth under Al treatment as those with a wild-type background, i.e., *ALMT1/ALMT1 NIP1;2/NIP1;2* (Table 1 and Additional file 1: Figure S3). In contrast, homozygous *almt1* and/or *nip1;2* plants in phenotypic groups B and C were more sensitive to Al compared with the plants in group A (Table 1 and Additional file 1: Figure S3). These results indicated that the wild-type alleles of *ALMT1* and *NIP1;2* were both completely dominant.

Although homozygous mutations of *almt1* or *nip1;2* caused significant root growth inhibition (Fig. 2 and Table 1), the effects of genotypic variation at one locus on the phenotypic expression of the other locus were quite different between *ALMT1* and *NIP1;2* (Table 1). For instance, under a homozygous *almt1/almt1* background (group B, Table 1), root growth was solely determined by the homozygous *almt1* mutant alleles regardless of the genotypic variation at the *NIP1;2* locus (Table 1 and Additional file 1: Figure S3). In contrast, under a homozygous *nip1;2*/*nip1;2* background, the degrees of root growth inhibition by Al were strongly affected by the genotypic variation at the *ALMT1* locus. For instance, plants with homozygous *almt1/almt1* alleles, i.e., the *almt1_nip1;2* double mutant plants, displayed greatly enhanced root-growth inhibition compared with those with one or two copies of the wild-type *ALMT1* allele in group C (Table 1 and Additional file 1: Figure S3). The fact that the homozygous *almt1* mutation at the *ALMT1* locus could mask/override the effects of genotypic variation at the *NIP1;2* locus indicates that there exist interactions between the *ALMT1* and *NIP1;2* loci where *ALMT1* is genetically epistatic to *NIP1;2*.

### Additive effects of *ALMT1* and *MATE* and epistatic relationship between *ALMT1* and *NIP1;2* in Al resistance

**Fig. 3:**
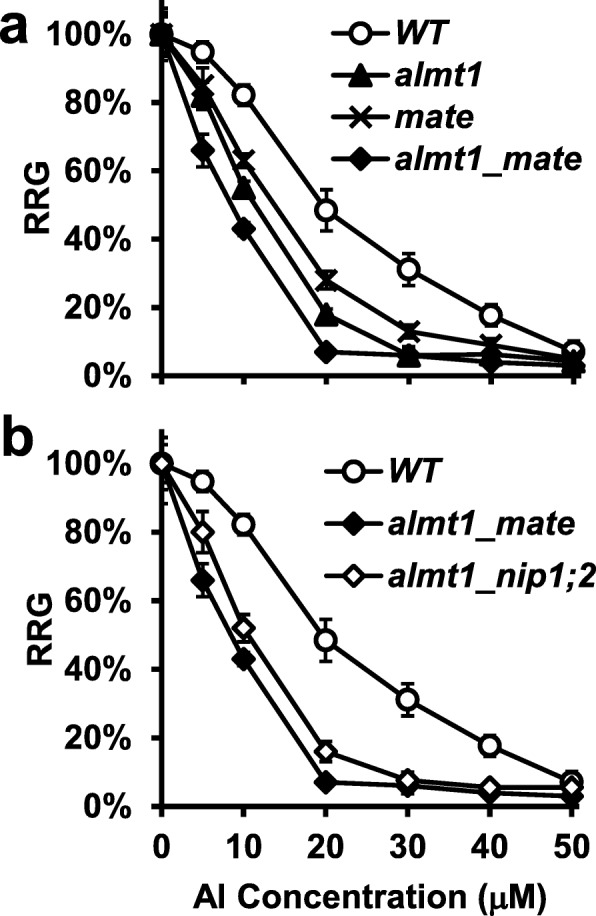
Relative root growth (RRG%) of *WT* and *almt1, mate* and *almt1_mate* mutants (**a**) and *WT* and *almt1_mate, almt1_nip1;2* double mutants (**b**). Seeds were germinated and grown in hydroponic solution (pH 4.3) supplemented with 0, 5, 10, 20, 30, 40, 50 μM of AlCl_3_ for 5 days. Data are means ± s.d. (*n* = 10)

In Arabidopsis, the Al-activated and ALMT1-facilated malate exudation from the root-tip region plays a major role in Al resistance, while the Al-activated and MATE-mediated citrate release from more mature root regions plays a smaller but significant role [24–26]. Although both the *almt1* and *mate* single mutants were more sensitive to a range of Al concentrations (0–50 μM) tested than was the WT, *almt1* consistently displayed significantly stronger root-growth inhibition than did the *mate* mutant (Fig. 3a and Additional file 1: Figure S2).

Compared with the *almt1* and *mate* single mutants, the *almt1_mate* double mutant showed significantly more severe root-growth inhibition phenotypes over the entire range of Al concentrations tested (Fig. 3a and Additional file 1: Figure S2). For instance, at 20 μM Al, root growth of *almt1_mate* was inhibited by 93%, whereas root growth of *almt1* and *mate* by 82 and 72%, respectively (Fig. 3a). Thus, the effects of *ALMT1* and *MATE* on Al resistance are additive, suggesting that ALMT1 and MATE function in different biochemical pathways, which is consistent with our previous observation that ALMT1 and MATE function independently in achieving overall Al resistance in Arabidopsis [25].

In contrast, the *almt1_nip1;2* double mutant did not display stronger mutant phenotypes than did the *almt1* single mutant (Fig. 2). Instead, root growth was comparable between *almt1* and *almt1_nip1;2* over the entire range of Al concentrations tested (Fig. 2).

### ALMT1-mediated root exudation of malate is independent of NIP1;2 function

**Fig. 4:**
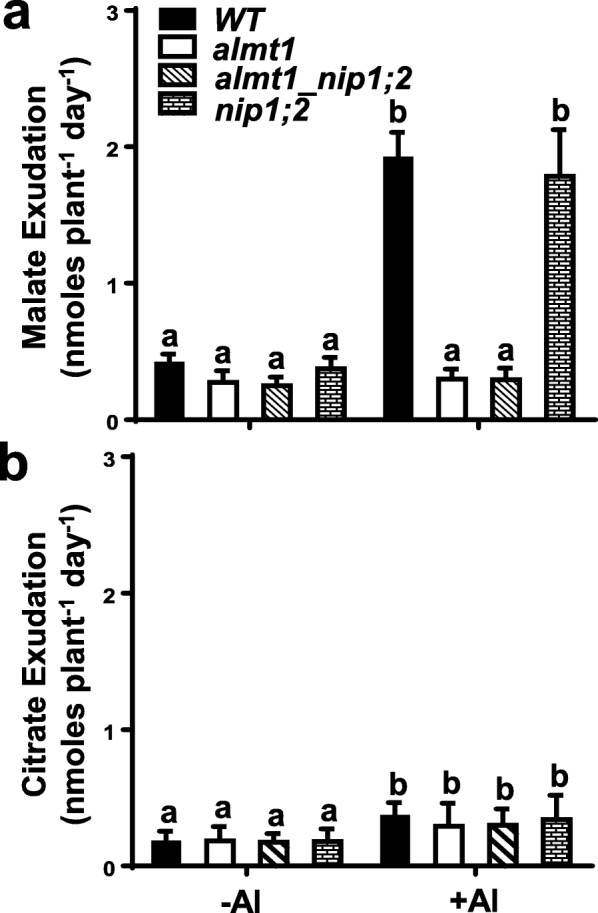
Root exudation of malate (**a**) and citrate (**b**). Here, 6-day-old seedlings of *WT*, *almt1, nip1;2* and *almt1nip1;2* were treated in 20 ml of exudation buffers (pH 4.3) supplemented without (−) or with (+) 50 μM AlCl_3_ for 2 days before the concentrations of malate and citrate in the exudation buffer were determined. Data are means ± s.d. of three biological replicates. *Letters* represent groups with significant differences (*P* ≤ 0.05)

To evaluate the effects of different genotypes on root organic acid exudation, Al-activated root exudation of malate and citrate was examined for *WT*, *almt1*, *nip1;2* and *almt1_nip1;2.* Under the control condition (−Al), comparable basal levels of root exudation of malate and citrate were observed among individual lines (Fig. 4a, b). Al exposure triggered releases of large and comparable amounts of malate from the roots of *WT* and the *nip1;2* mutant (Fig. 4a). In contrast, both *almt1* and *almt1_nip1;2* lacked detectable Al-activated root malate exudation (Fig. 4a). These results indicate that Al-activated malate exudation from the root is mainly facilitated by the ALMT1 malate transporter in Arabidopsis and the Al-activated and ALMT1-mediated root malate exudation is independent of the NIP1;2 function.

Compared with root malate exudation, Al exposure also triggered smaller, but significant, increases in citrate exudation from the root (Fig. 4b). In contrast, no significant differences were observed in the amounts of citrate in the root exudates from all lines examined upon Al exposure (Fig. 4b). These results indicate that the Al-activated and MATE-facilitated root citrate exudation is independent of the ALMT1 and NIP1;2 functions in Arabidopsis (Fig. 4b). Thus, the phenotypes of organic acid exudation of the *almt1_nip1;2* double KO line resemble those of the *almt1* mutant, but not the *nip1;2* mutant.

### ALMT1 functions upstream of NIP1;2 in the process of Al removal from the root cell wall

**Fig. 5:**
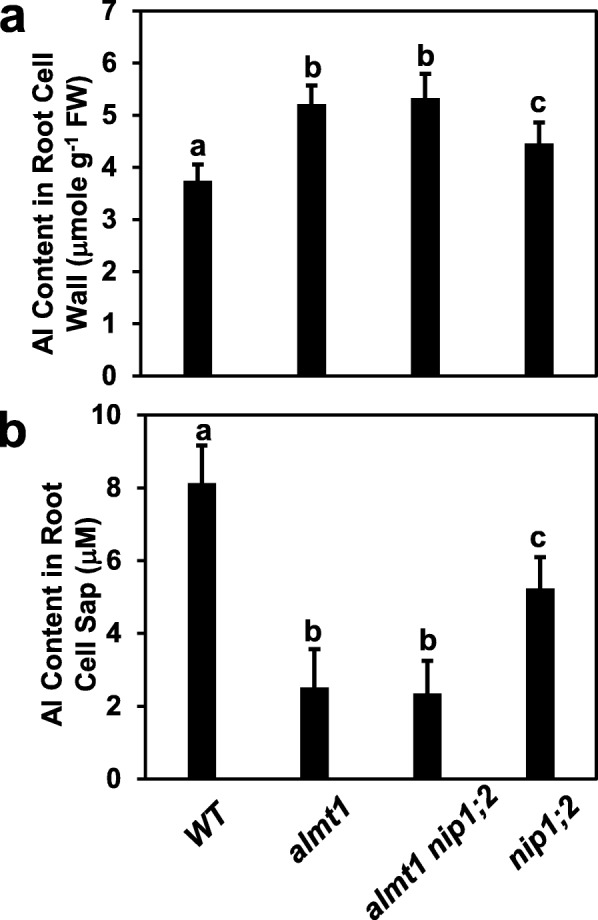
Aluminum content in the root cell wall (**a**) and the root cell sap (**b**). Seven-day-old seedlings of WT, *almt1, nip1;2* and *almt1_nip1;2* were exposed to 50 μM AlCl_3_ (pH 4.3) for 2 days. Al concentrations in the root cell wall (**a**) and the root cell sap (**b**) were determined by ICP-MS. Data are mean ± s.d. of three biological replicates from three Magenta boxes. *Letters* represent groups with significant differences (*P* ≤ 0.05)

To test the relationship between *ALMT1* and *NIP1;2* in the processes of Al removal from the root cell wall, Al contents in the root cell wall and cell sap were measured for the *WT*, *almt1*, *nip1;2* and *almt1_nip1;2* plants treated with 50 μM AlCl_3_ at pH 4.3 for 2 d (Fig. 5). Compared with the *WT* plants, the *almt1* and *nip1;2* plants accumulated significantly higher and lower concentrations of Al in the root cell walls (Fig. 5a) and root cell sap (Fig. 5b), respectively. These results confirmed that both ALMT1-mediated malate releases and a functional NIP1;2 are required for Al removal from the root cell wall into the root cytosol [19]. However, the *almt1* mutant also accumulated significantly higher and lower concentrations of Al in the root cell wall (Fig. 5a) and the root cell sap (Fig. 5b), respectively, compared with the *nip1;2* mutant. These results suggest that ALMT1-depenedent but NIP1;2-indenpent processes are present for Al removal from the root cell wall in Arabidopsis.

The Al concentrations in the root cell wall (Fig. 5a) and root cell sap of the *almt1_nip1;2* double mutant (Fig. 5b) were comparable with those of the *almt1* single mutant, which were significantly different from those in the *nip1;2* single mutant. These results indicate that *ALMT1* is genetically epistatic to *NIP1;2* in the biochemical pathway leading to Al removal from the root cell wall into the root symplasm in Arabidopsis.

### Externally supplied malate partially restored NIP1;2-facilitated Al uptakes from the root cell wall in *almt1* but not in *almt1_nip1;2*

**Fig. 6:**
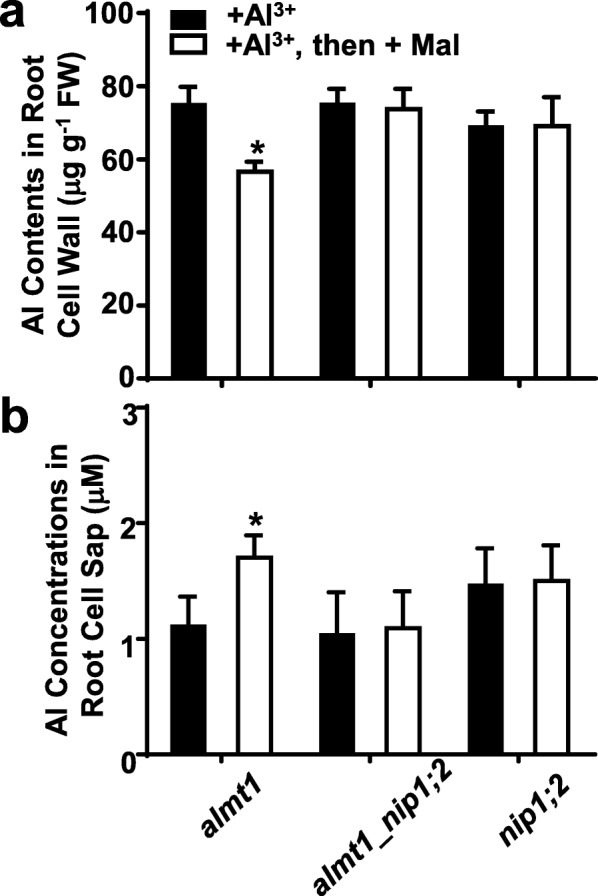
Effects of externally supplied malate in NIP1;2-mediated Al uptake in the *almt1, almt1_nip1;2* and *nip1;2* lines. Here, 7-day-old seedlings were pretreated with AlCl_3_ (pH 4.3) for 8 h, washed three times with 0.5 mM CaCl_2_, and then treated with 200 μM malate (−Al) for 8 h. **a** Al concentrations in the root cell wall and **b** root cell sap were determined by ICP-MS. Data are mean ± s.d. of three sample replicates from three Magenta boxes. *, significant differences (*P* ≤ 0.05) between the – and the + malate treatment

To evaluate the effects of externally supplied malate on Al uptakes from the root cell wall for *almt1*, *nip1;2* and *almt1_nip1;2*, plants were treated with 50 μM AlCl_3_ (pH 4.3) for 8 h, allowing Al to get into and be retained in the root cell walls (Fig. 6a) [19], followed by addition of 0 or 200 μM malate for another 8 h.

Between these two treatments, no statistically significant differences in Al contents in the root cell wall (Fig. 6a) and the root cell sap (Fig. 6b) were observed in the *nip1;2* single mutant and the *almt1_nip1;2* double mutant. In contrast, in *almt1*, compared with those under the Al treatment alone, external supplementation of malate after Al treatment led to significantly decreased Al concentrations in the root cell wall (Fig. 6a) and significantly increased concentrations in the root cell sap (Fig. 6b). These results indicate that even though the *almt1* mutant has a functional NIP1;2 transporter [19], the presence of malate in the root cell wall is essential for NIP1;2-facilitated Al removals from the root cell wall. Thus, the ALMT1-mediated releases of malate to the root cell wall function in an earlier step in the NIP1;2-facilitated process for Al uptakes from the root cell wall to the root cytosol.

## Discussion

### *ALMT1* is genetically and functionally epistatic to *NIP1;2*

In Arabidopsis, coordinated activity of ALMT1 and NIP1;2 is required for NIP1;2-facilitated Al removal from the root cell wall into the root symplasm and subsequent root-to-shoot Al translocation, which are critical steps in the internal detoxification mechanism [19, 32, 35]. In this process, first, the Al^3+^ ions enter the root apoplast from the rhizosphere and then activate the PM-localized ALMT1 transporter, leading to malate exudation from the root tip cell into the root apoplast and rhizosphere [24–26]. In the root apoplast, the released malate interacts with the Al^3+^ ions to form Al-Mal complexes, which are subsequently transported from the root cell wall into the root cytosol by the PM-localized NIP1;2 [19]. As NIP1;2 transports the Al-Mal complex, but not the Al^3+^ ion, the ALMT1-mediated malate exudation into the root cell wall is required for the formation of Al-Mal complexes in the root apoplast and the subsequent NIP1;2-facilitated Al removal from the root cell wall (Fig. 5) [19]. Thus, ALMT1 plays a key role in both the exclusion mechanism, via facilitating malate exudation to the rhizosphere to chelate toxic Al^3+^ ions, and the internal detoxification mechanism, through facilitating the NIP1;2-mediated removal of the Al-Mal complex from the root cell wall and translocation from the root to the shoot [19].

Phenotypic examination of root growth indicated that the *almt1*_*mate* double mutant was more sensitive to Al than was either of the *almt1* or *mate* single mutants, indicating that the effects of *almt1* and *mate* mutations are additive (Fig. 3a). In contrast, no additive or synergistic effects were observed between the *almt1* and *nip1;2* mutations (Fig. 2 and Additional file 1: Figure S1). Therefore, our root-growth experimental conditions could distinguish the additive effect of *ALMT1* and *MATE* from the epistatic relationship between *ALMT1* and *NIP1;2*. Further examination of Al-activated root organic-acid exudation (Fig. 4) and NIP1;2-facilitated Al removal from the root cell wall (Fig. 5) also indicated that the phenotypes of the *almt1_nip1;2* double KO mutant resembled those of *almt1*, but not *nip1;2*. Moreover, externally supplied malate could partially compensate the loss of the ALMT1-mediated malate exudation for the NIP1;2-facilitated function in the *almt1* mutant (Fig. 6). Taken together, these results indicate that ALMT1 and NIP1;2 function in a single biochemical pathway, where ALMT1 functions upstream of NIP1;2. The fact that the *almt1* mutant is more sensitive to Al toxicity than was the *nip1;2* mutant (Fig. 2) also indicates that *ALMT1* plays a larger role in contribution to overall Al resistance than does *NIP1;2* in Arabidopsis.

### The significance of the epistatic relationship between ALMT1 and NIP1;2 in Al tolerance for the non-accumulating Arabidopsis

On acid soils, most plants, i.e., the so-called non-accumulators, limit the uptake of Al from the soil and accumulate no more than 0.2 mg Al g^− 1^ dry weight of the plant [32]. In contrast, a few Al accumulator plant species can accumulate much higher concentrations of Al in the shoot/leaf. For instance, hydrangea (*Hydrangea macrophylla*) plants can accumulate up to 3 mg Al g^− 1^ dry weight of the plant [32], while buckwheat (*Fagopyrum esculentum*) plants 1.7 mg Al g^− 1^ dry weight without showing any sings of toxicity [38, 39].

In both the accumulating and non-accumulating plant species, overall Al resistance can be achieved by the exclusion and the internal tolerance mechanisms [15, 32, 40]. However, these two mechanisms are not mutually exclusive but they are coordinately functioned as in the case of the ALMT1- and NIP1;2-mediated Al tolerance in Arabidopsis [19, 35].

Arabidopsis is a non-accumulating species for Al. Therefore, Al tolerance in Arabidopsis is mainly dependent on the exclusion mechanism via the ALMT1- and MATE-mediated root exudation of malate and citrate, respectively, to the rhizosphere and the root apoplast where organic acids chelate the Al^3+^ ions [24–26]. As ALMT1 facilitates the release of a large amount of malate from the root tip region [26], a major target for Al toxicity [12, 13, 41, 42], ALMT1 makes a significantly larger contribution to overall Al resistance in Arabidopsis than MATE [25, 26].

In the root apoplast, the released malate anions chelate the Al^3+^ cations and thus reduce the concentration of free Al^3+^ cations, which minimizes the harmful interactions of these cations in the cell wall. However, the simple binding of Al and malate in the apoplast of the root cell is not enough to provide full protection against Al toxicity [19]. Instead, the Al-Mal complexes in the root apoplast need to be removed for achieving higher degrees of Al tolerance [19].

Here, we further demonstrate that ALMT1 is genetically and functionally epistatic to NIP1;2. Such an epistatic relationship allows the Arabidopsis plant to be protected by the exclusion mechanism first, which effectively blocks the entry of toxic Al^3+^ cations into the root cell, including the root cell wall, before the NIP1;2-facilitated internal detoxification mechanism, i.e., uptakes of Al from the root cell wall, starts to function. Without the establishment of such an epistatic relationship between the exclusion and internal detoxification mechanisms, high levels of Al could be accumulated in the plant, which is harmful to the non-accumulator like Arabidopsis.

Therefore, for the non-accumulating plant species, a dominant exclusion mechanism is essential for Al resistance via continuously preventing the entry of Al to the root cell. After the exclusion mechanism is established, the internal detoxification mechanism will step in and play a secondary and scavenging role for removing the toxic Al^3+^ ions from the root apoplast and cytosol for further sequestration into the vacuole of the root cell and/or for translocation from the root to less sensitive shoot.

In contrast, for the accumulating plants to take up large amounts of Al^3+^ ions from the root and accumulate them in the shoot, the exclusion mechanism must be suppressed. Interestingly, Al-activated release of oxalic acid is required for the protection of root growth and function for young seedlings of buckwheat, an Al accumulator, under Al stresses [43]. Thus, it is likely that at the early developmental stage, the exclusion mechanism is also essential for Al accumulators to withstand the initial shocks of Al toxicity. Once the internal detoxification mechanism is established at later developmental stages, the exclusion mechanism will be suppressed or discontinued to function, allowing the accumulation of large amounts of Al in the shoot via the internal detoxification mechanism.

### Essential roles of the root cell wall in overall Al resistance in Arabidopsis

As mentioned above, the significance of the epistatic relationship between the exclusion and internal detoxification mechanisms in Arabidopsis lies in that the ALMT1-mediated exclusion mechanism provides a shield/barrier for the root against the toxic Al^3+^ ions in the rhizosphere before the NIP1;2-facilitated internal detoxification mechanism is allowed to function. However, the pivotal roles of the root cell wall are less well recognized in this system.

The root cell wall is highly negatively charged and as a result, Al^3+^ cations in the rhizosphere can freely enter and be retained in the root apoplast at low pH (< 5.0) [19]. The Al-activated and ALMT1-mediated malate release to the rhizosphere results in the formation of Al-Mal complexes in the rhizosphere. As the Al-Mal complex is unable to enter the root cell wall [19], with a functional ALMT1-mediated malate exudation to the rhizosphere, it is the root cell wall that acts as a shield/barrier that separates the Al in the rhizosphere from the root symplasm. Furthermore, the presence of such a shield ensures that only the Al-Mal complex in the root cell wall, but not in the rhizosphere, is accessible to the NIP1;2 transporter localized to the PM of the root cell. In conclusion, the root cell wall plays pivotal roles in the establishment of the exclusion mechanism as well as the epistatic relationship between the exclusion and the internal detoxification mechanisms for overall Al resistance and for the prevention of over-accumulation of Al in Arabidopsis plants.

## Conclusion

Here, we demonstrate that ALMT1 is genetically epistatic to NIP1;2 for achieving coordinated functions between an exclusion and an internal tolerance mechanism and overall Al resistance in the non-accumulating Arabidopsis plant. This elegant system ensures that an exclusion mechanism is established before an internal tolerance mechanism steps in to achieve overall Al resistance in the non-accumulating Arabidopsis. The root cell wall plays indispensable roles in implementation of the Al exclusion mechanism and the establishment of an epistatic relationship between the ALMT1-mediated exclusion mechanism and the NIP1;2-facilitated internal detoxification mechanism. These findings further expand our knowledge about how overall Al resistance is achieved in plants.

## Methods

### Materials and culture conditions

Arabidopsis T-DNA insertion lines *nip1;2–1* (SALK_126593), *nip1;2–2* (SALK_147353), *nip1;2–3* (SALK_076128), *mate* (SALK_081671) and *almt1* (SALK_009629C) as well as WT (*Col-0*; CS60006) were acquired from the Arabidopsis Biological Resource Center (https://abrc.osu.edu/). The *almt1_nip1;2* double mutant was generated by crossing *almt1* and *nip1;2–3* (*nip1;2*) single mutants, followed by selection of F2 plants with homozygous T-DNA insertions at both the *ALMT1* and *NIP1;2* loci by PCR-based genotyping [19]. The *almt1_mate* double mutant was generated previously [25].

Primer sequences for genotyping the *ALMT1* locus were 5′-CGCAGCTGCACATATATCACA-3′ (*ALMT1* gene specific primer) and 5′-GCTGTTGCCCGTCTCACTGGTG-3′ (T-DNA left border primer) for detection of the T-DNA insertion; and 5′-CGCAGCTGCACATATATCACA-3′ and 5′-CGAAGTGCAACGCACCACTA-3′ for amplification of the sequence encompassing the T-DNA insertion region. Primer sequences for genotyping the *NIP1;2* locus for *nip1;2–3* were 5′-GCTCGCATCTAGATCCTAAT-3′ (*NIP1;2* gene specific primer) and 5′-GCTGTTGCCCGTCTCACTGGTG-3′ (T-DNA left border primer) for detection of the T-DNA insertion; 5′- GCTCGCATCTAGATCCTAAT-3′ and 5′-CGAAGTGCAACGCACCACTA-3′ for amplification of the sequence encompassing the T-DNA insertion region. The positions of the T-DNA insertions as well as the primers listed above were depicted in Additional file 1: Figure S4.

The PCR mixture (25 μL) contains the following reagents: 100 ng gDNA template, 1X Green GoTaq® Reaction Buffer (1.5 mM MgCl_2_) (Promega), 0.2 mM each dNTP, 0.5 μM upstream primer, 0.5 μM downstream primer, GoTaq DNA polymerase (Promega). The PCR reactions were conducted with a T100 Thermal Cycler (BioRad) with the following thermal cycling conditions: 1 cycle of 95 °C for 2 min; 25 cycles of 95 °C for 1 min, 55 °C for 1 min, and 72 °C for 2 min; final extension at 72 °C for 5 min.

For growth experiments, seeds of the wild type, individual mutant lines or the F2 population from the cross of *almt1* and *nip1;2* were surface-sterilized and cold stratified at 4 °C in the dark for 3 days for synchronization of germination. Seeds were subsequently sown onto a 250 μM polypropylene mesh in a Magenta box containing a hydroponic growth solution, supplemented with 2.0 mM Homo-PIPES to maintain pH at 4.3. The hydroponic solution consisted of the following macronutrients in mM: MgCl_2_, 3.0; (NH_4_)_2_SO_4_, 0.25; Ca (NO_3_)_2_, 1.0 M; KCl, 2.0; CaCl_2_, 2.75; KH_2_P0_4_, 0.18; and the following micronutrients in μM: H_3_BO_3_, 50.0; MnSO_4_, 10.0; CuSO_4_, 0.5; ZnSO_4_, 2.0; Na_2_MoO_4_, 0.1; CoCl_2_, 0.1; 1% sucrose. Plants were grown in a growth chamber (Pervival, Model I-36LLVL) with 23 °C temperature, 65% humidity and light intensity of 100 μmol photons m^2^/s by cool-white fluorescent tubes (GE) and 16-h photoperiod.

For evaluating Al sensitivity, seeds of the WT and individual mutant lines, i.e., *nip1;2*, *almt1*, *almt1_mate* and *almt1_nip1;2*, were germinated and grown in the above-mentioned hydroponic solution (pH 4.3) supplemented with 0, 5, 10, 20, 30, 40 or 50 μM of AlCl_3_ for 7 days. Relative root growth (RRG%) was calculated according to the following formula: RRG% = root growth (mm) of individual plants under Al treatment/mean root growth (mm) under the control (−Al) condition.

For phenotyping and genotyping the F2 individuals derived from the cross of *almt1* and *nip1;2*, F2 seeds were germinated and grown in the hydroponic solution (pH 4.3) supplemented with 20 μM AlCl_3_ in Magenta boxes (~ 120 seed in each box) for 7 days. Root length (mm) of 215 randomly selected plants was measured before the plants were transferred to the soil for growth for 2 weeks. Then, DNAs were extracted from leaves of individual F2 plants. T-DNA insertions at the *ALMT1* and *NIP1;2* loci were evaluated by PCR followed the procedures mentioned above. Based on the genotypes at the *ALMT1* and *NIP1;2* loci, the F2 population could be classified into nine distinct genotypic combinations/groups (Table 1; Additional file 1: Figure S3). Ten plants were randomly selected from each of the nine genotypic groups for calculation of the mean root growth (mm) of the group (Table 1; Additional file 1: Figure S3). The root growth data were also used for performing Fisher’s least significant difference (LSD) tests to distinguish statistically different phenotypic groups in the F2 population (Table 1; Additional file 1: Figure S3).

### RNA isolation and real-time RT-qPCR

For gene expression analysis, ~ 500 seeds (~ 10 mg) were germinated in the above-mentioned control hydroponic solution (−Al) in a Magenta box for 6 days. Then, seedlings were transferred to a fresh hydroponic solution (pH 4.3) containing 20 μM AlCl_3_ and treated for 24 h before the root samples were collected. Three replicates (Magenta boxes) were included for each of the *WT*, *nip1;2*, *almt1* and *almt1_nip1;2* lines.

Total RNAs were extracted from the roots using an RNeasy Mini Kit (Qiagen) following the manufacturer’s instructions. First-strand cDNA was synthesized in a reaction cocktail containing 1X reaction buffer, 5 μg DNaseI-digested total RNA, 2.5 μM of random oligos, 1 mM of each dNTP, 5 μL of SuperScript III reverse transcriptase (Thermal Scientific, Inc.) in a total volume of 100 μL. The reaction was performed at 37 °C for 90 min, followed by heating at 72 °C for 10 min. Subsequently, 2 μL of RNase H (Thermal Scientific, Inc.) was added to each RT sample for 1.5-h incubation at 37 °C. The synthesized samples were stored at -20 °C until use.

Real-time RT-qPCR was performed on a 7500 Fast Real-Time PCR System (Thermal Scientific, Inc.). Concentrations of each of cDNA samples were adjusted to 1 μg/μL. Each real-time PCR reaction contained 2 μL of diluted cDNA sample, 10 μL of 2X Power SYBR Green PCR Master Mix (Thermal Scientific, Inc.), 0.15 μM primer (forward and reverse each) in 20 μl reaction volume. Three technical replicates were included for each cDNA sample. The real-time PCR cycling conditions were 95 °C for 3 min; 39 cycles of 95 °C for 10 s, 60 °C for 30 s, plate reading; 1 cycle of 65 °C for 30 s; 60 cycles of 65 °C for 5 s (+ 0.5 °C/cycle, ramp 0.5 °C/sec), plate reading.

The sequences of optimal gene-specific real-time RT-qPCR primers were *NIP1;2*, 5′- GGTTCGATATACTGATAAGCCA-3′ and 5′-GATACAACTTAACCTCCGATGAC-3′ (137 bp amplicon); *ALMT1*, 5′-TTCCCGATTCCGAGCTCATT-3′ (located in exon 5 and exon 6 junction) and 5′-CTCAGATTTTCAGATCCCAGTGGAC-3′ (80 bp amplicon); *18S* rRNA (endogenous calibrator gene), 5′-CGCTATTGGAGCTGGAATTACC-3′, 5′-AATCCCTTAACGAGGATCCATTG-3′ (71 bp amplicon). Gene structure, locations of the real-time PCR primers for corresponding genes were depicted (Additional file 1: Figure S5). In all real-time PCR amplifications mentioned below, a single peak of the dissociation curve was observed for each of the real-time RT-qPCR primer pairs, indicating that the primers were highly specific for the target genes.

To construct standard curves for the *NIP1;2*, *ALMT1* and *18S rRNA* amplifications, a serial dilutions (5x) of a cDNA sample (1 μg/μl) prepared from total WT RNA were prepared and subject to real-time PCR thermal cycling as mentioned above. Standard curves for the target genes (*ALMT1* and *NIP1;2*) and the endogenous control gene (*18S rRNA*) were plotted as the log ng cDNA (six logs or dilutions included, three technical replicates for each dilution) vs. C_T_ values of the corresponding samples. The equation for the standard curves was y = mx + b, where y was C_T_, x was log ng of the cDNA sample, m the slope of standard curve line and b the y-intercept of the standard line. For the standard curves of the *NIP1;2*, *ALMT1* and *18S rRNA* amplification tested, m was ~ − 3.3 and R^2^ > 0.99, indicating high efficiency of the primers for PCR amplification.

Real-time qPCR samples for testing the expression of a known gene (i.e., *ALMT1*, *NIP1;2* or *18S rRNA*) were put in a same 384-well plate together with the standard curve samples of the corresponding gene. The qPCR thermal cycling conditions were as mentioned above. The quantity of the real-time qPCR amplicons of the known gene were calculated for each sample based on its CT value and the standard curve of the corresponding known gene. Relative gene expression was calculated as the quantity of the target genes (i.e., *ALMT1* or *NIP1;2*) divided by the quantity of the *18S rRNA* gene of the same cDNA sample.

### Detection of organic acid exudation from roots

Surface-sterilized seeds (~ 2–3 mg) from each line were germinated in Magenta boxes containing the sterile hydroponic growth solution (pH 4.3) for 6 days, and then the seedlings were transferred to 20 ml of filter-sterilized exudation solutions (pH 4.3) with or without 50 μM Al^3+^ in a sterile Petri dish for 2 days. The exudation solution consisted of the following macronutrients in μM: MgCl_2_, 275; CaCl_2_, 275; KCl, 275; Ca (NO_3_)_2_, 33.4; MgSO_4_, 33.4; K_2_SO_4_,16.7; and the following micronutrients in μM: H_3_BO_3_, 50.0; MnSO_4_, 10.0; CuSO_4_, 0.5; ZnSO_4_, 2.0; Na_2_MoO_4_, 0.1; CoCl_2_, 0.1; and 1% sucrose, supplemented with 3.0 mM Homo-PIPES (pH 4.3). Then, the exudation solutions were collected and the numbers of plants were counted. To remove Al^3+^ and other inorganic anions, the exudation solutions were treated with anionic and cationic chromatography columns. Subsequently, the eluate was concentrated to dryness using a rotary evaporator at 40 °C. The residue was re-dissolved in 1 ml of Milli-Q water. Malate and citrate concentrations were then measured according to the enzymatic method previously described [22].

### Root cell sap and cell wall preparation and Al determination

Arabidopsis lines were firstly germinated and grown in the hydroponic solution (pH 4.3) for 7 days, then treated in a fresh hydroponic solution (pH 4.3) supplemented with 50 μM AlCl_3_ for 2 d. After the treatment, the roots were cut and washed three times with deionized water. The cut root samples were centrifuged at 3000 rpm for 10 min at 4 °C in an Ultra free-MC Centrifugal filter unit (Millipore) to remove the apoplastic solution, and frozen in a − 80 °C freezer overnight. The frozen root samples were de-frozen at room temperature, and then centrifuging at 13,000 rpm for 10 min to separate the root cell sap solution from the residual cell wall. The cell wall sample was washed with 70% ethanol three times and then digested in 1 mL of 2 M HCl for at least 24 h with gentle shaking. Al contents in the symplastic solution and cell wall extract were determined by inductively coupled plasma mass spectrometry (ICP-MS).

For testing the effects of sequential Al^3+^ and malate treatment on Al accumulation, ~ 150 7-d-old seedlings of the WT, *almt1, nip1;2–3* and *almt1_nip1;2* lines were pretreated with the hydroponic solution (pH 4.3) supplemented with 50 μM AlCl_3_ for 8 h. The samples were then washed three times with 0.5 mM CaCl_2_ and treated in hydroponic solutions (pH 4.3) supplemented with or without 200 μM malate for 8 h. Aluminum concentrations in cell sap and cell wall were measured as mentioned above. Three biological replicates (Magenta boxes) with the same setting were prepared for each plant line and each treatment.

## Supplementary information

**Additional file 1 Figure S1.** Seeds of *WT* and *nip1;2–1, nip1;2–2* and *nip1;2–3* mutants were germinated and grown in hydroponic solution (pH 4.3) supplemented with 20 μM of AlCl_3_ for 5 days. **Figure S2.** Seeds of *WT, almt1, mate, nip1;2* and *almt1_mate, almt1_nip1;2* double mutants were germinated and grown in hydroponic solution (pH 4.3) supplemented without (−Al) or with (+Al) 20 μM of AlCl_3_ for 5 days. **Figure S3.** Root growth of different genotypes of the F2 population. **Figure S4.** Gene structure, positions of T-DNA insertions and PCR primers of *ALMT1* and *NIP1;2.***Figure S5.** Gene structure, position and size of real-time RT-PCR amplicon for *ALMT1*, *NIP1;2* and *18S rRNA*.

## Data Availability

The datasets used and analyzed during the current study are available from the corresponding author on reasonable request.

## Abbreviations

Al-Mal: Aluminum-malate
ALMT: Al-activated malate transporter
KO: Knockout
MATE: Multidrug and toxic compound extrusion
NIP: Nodulin 26-like intrinsic protein
RRG: Relative root growth
