# Supplementary material for: An exclusion mechanism is epistatic to an internal detoxification mechanism in aluminum resistance in Arabidopsis
Source: BMC Plant Biol. 2020 Mar 18;20:122. doi: 10.1186/s12870-020-02338-y (PMC7079475; doi:10.1186/s12870-020-02338-y)
Supplement: Supplementary file 1 — Additional file 1 Figure S1. Seeds of WT and nip1;2–1, nip1;2–2 and nip1;2–3 mutants were germinated and grown in hydroponic solution (pH 4.3) supplemented with 20 μM of AlCl3 for 5 days. Figure S2. Seeds of WT, almt1, mate, nip1;2 and almt1_mate, almt1_nip1;2 double mutants were germinated and grown in hydroponic solution (pH 4.3) supplemented without (−Al) or with (+Al) 20 μM of AlCl3 for 5 days. Figure S3. Root growth of different genotypes of the F2 population. Figure S4. Gene structure, positions of T-DNA insertions and PCR primers of ALMT1 and NIP1;2.Figure S5. Gene structure, position and size of real-time RT-PCR amplicon for ALMT1, NIP1;2 and 18S rRNA. [file 12870_2020_2338_MOESM1_ESM.pdf]

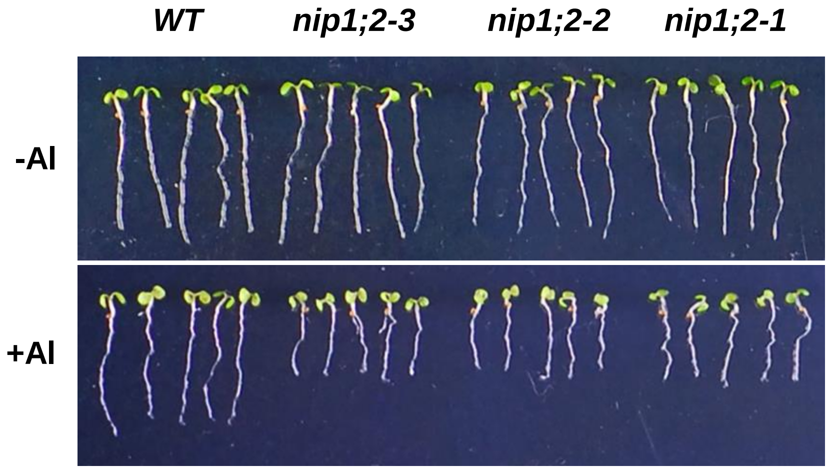

Figure S1. Seeds of *WT* and *nip1;2-1, nip1;2-2* and *nip1;2-3* mutants were germinated and grown in hydroponic solution (pH 4.3) supplemented with 20 μM of $AlCl_3$ for 5 days. For taking the photos, five 5-day-old seedlings were randomly selected from each line for each treatment.

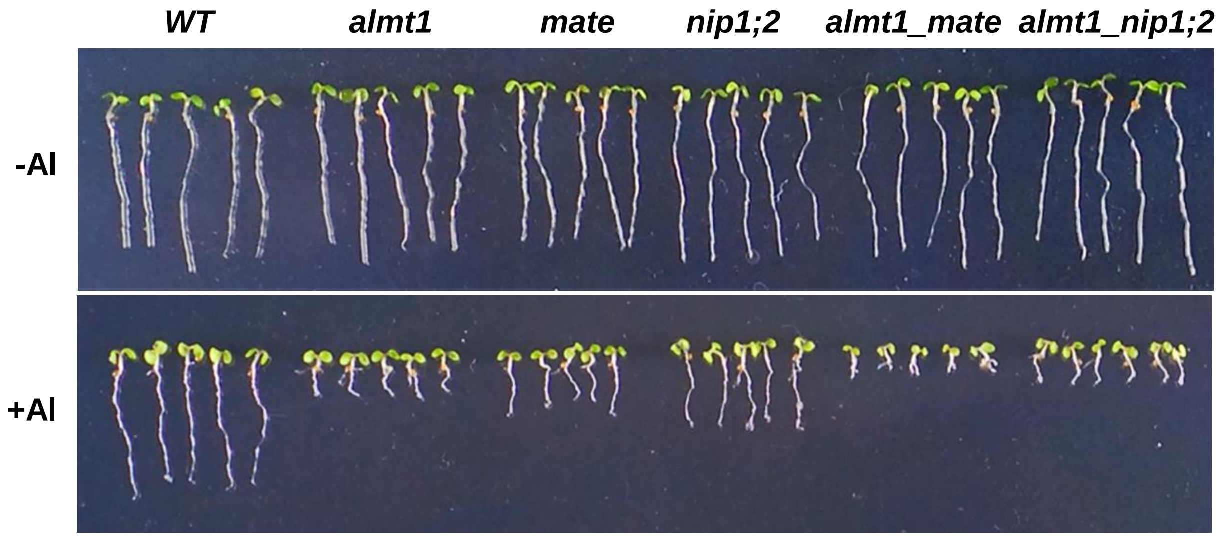

Figure S2. Seeds of *WT, almt1, mate, nip1;2* and *almt1_mate, almt1_nip1;2* double mutants were germinated and grown in hydroponic solution (pH 4.3) supplemented without (-Al) or with (+Al) 20 μM of $AlCl_3$ for 5 days. For taking the photos, five 5-day-old seedlings were randomly selected from each line for each treatment.

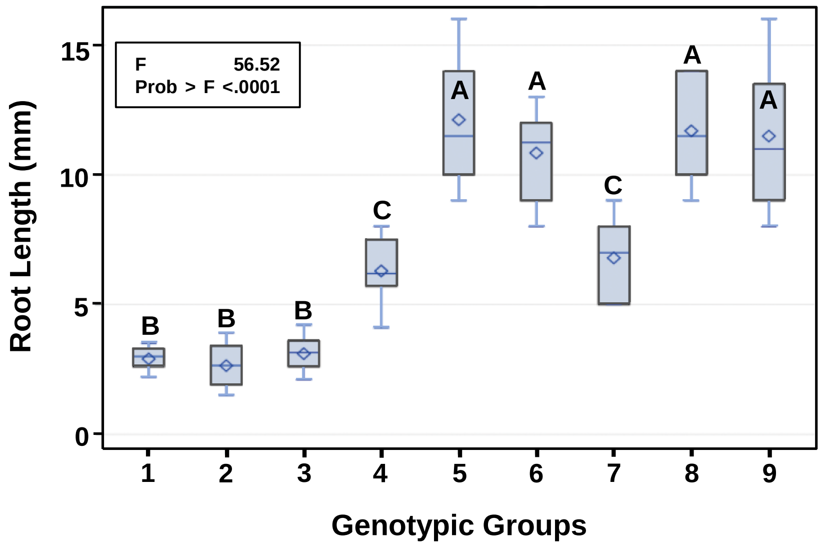

Figure S3. Root growth of different genotypes of the F2 population. F2 seedlings were germinated and grown in a hydroponic solution supplemented with 20 μM $AlCl_3$ (pH 4.3) for 7 days. The F2 population has nine genotypes: ***1)*** *almt1/almt1 nip1;2/nip1;2*; ***2)*** *almt1/almt1 nip1;2/NIP1;2*; ***3)*** *almt1/almt1 NIP1;2/NIP1;2*; ***4)*** *almt1/ALMT1 nip1;2/nip1;2*; ***5)*** *almt1/ALMT1 nip1;2/NIP1;2*; ***6)*** *almt1/ALMT1 NIP1;2/NIP1;2*; ***7)*** *ALMT1/ALMT1 nip1;2/nip1;2*; ***8)*** *ALMT1/ALMT1 nip1;2/NIP1;2*; ***9)*** *ALMT1/ALMT1 NIP1;2/NIP1;2.* Data are mean root growth (mm) ± s.d. (N = 10). *Letters* represent groups with significant root length difference ($P \leq 0.05$) as determined by Fisher's LSD test.

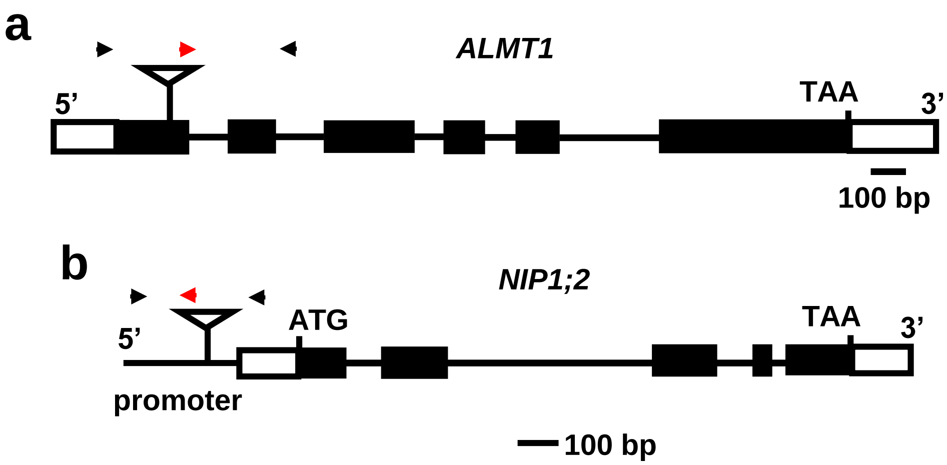

Figure S4. Gene structure, positions of T-DNA insertions and PCR primers of *ALMT1* (**a**) and *NIP1;2* (**b**). Box, exon; horizontal line, intron; closed box, coding sequence; open box, non-coding sequence; triangle, T-DNA insertion; black arrow, gene specific PCR primer flanking the T-DNA insertion; read arrow, primer in the left border of the T-DNA insertion.

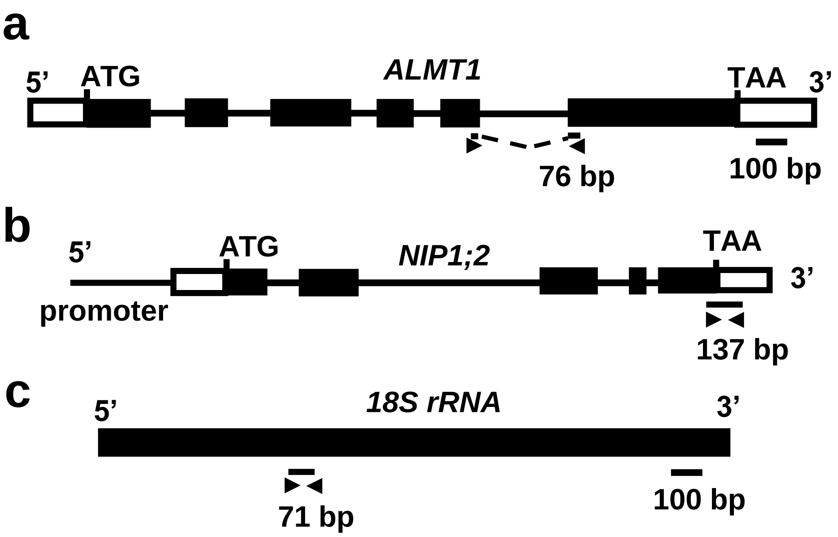

Figure S5. Gene structure, position and size of real-time RT-PCR amplicon for *ALMT1* (**a**), *NIP1;2* (**b**) and *18S rRNA* (**c**). Arrows represent the real-time RT-PCR primers.
